# Ovarian Sertoli Cell Tumor with Immature Prepubertal-like Sertoli Cell Component: A Case Report and Literature Review

**DOI:** 10.3390/medicina58111638

**Published:** 2022-11-13

**Authors:** Diana Bužinskienė, Evelina Šidlovska, Gabija Vilutytė

**Affiliations:** 1 Faculty of Medicine, Vilnius University, LT-03101 Vilnius, Lithuania; 2 Center of Obstetrics and Gynecology, Institute of Clinical Medicine, Faculty of Medicine Vilnius University, LT-08661 Vilnius, Lithuania; 3 National Center of Pathology, Affiliate of Vilnius University Hospital Santaros Klinikos, LT- 08406 Vilnius, Lithuania

**Keywords:** pure Sertoli cell tumor, sex chord tumor, non-epithelial ovarian cancer

## Abstract

The Sertoli cell tumor of the ovary is a rare ovarian tumor with non-specific symptoms. According to the literature, endocrine manifestations occur in two-thirds of patients, but testosterone production is extremely rare. Typically, it is a unilateral benign tumor of the ovary that most commonly presents in adolescents and young women of childbearing potential. We report a 29-year-old patient, previously diagnosed to have polycystic ovarian syndrome, who presented with complaints of amenorrhea for the past three years. A transvaginal ultrasound scan revealed polycystic structure ovaries and a solid cystic formation of 32 × 31 mm size with strong blood flow in the left ovary. The laboratory tests reported an elevated testosterone level. During laparoscopic surgery, a solid, yellowish tumor was removed and the left ovary was resected. Histological examination revealed a left ovary Sertoli cell tumor with an immature prepubertal-like Sertoli cell component. Following surgery, the serum testosterone levels returned to normal and the menstrual cycle became regular. Due to the substantially low incidence of ovarian Sertoli cell tumors, information on their clinical behavior, morphologic spectrum, optimal management, and prognosis is limited. They are characterized by a wide variety of clinical manifestations, treated surgically, and, if diagnosed at an early stage, have good prognosis. We emphasize the extraordinarily rare clinical presentation of this case report.

## 1. Introduction

Sertoli cell tumors occur in both gonads, but are more common in the testis [1]. Ovarian Sertoli cell tumors are a rare subgroup of sex-chord stromal tumors of the ovary; together with Sertoli–Leydig cell tumors and Leydig, cell tumors they account for less than 1% of all ovarian tumors [2].

The histogenesis of Sertoli tumors of the ovary is entirely hypothetical; it is thought that they arise from cells within the ovary that have retained their potential to differentiate in appearance and function toward a Sertoli cell [3]. Typically, they are unilateral benign tumors of the ovary that most commonly present in adolescents and young women of childbearing potential [4]. Sertoli cell tumors may be diagnosed incidentally as nonfunctioning asymptomatic ovarian masses or present as hormone-producing tumors associated with hormonal hyperactivity [4,5]. The tumor usually produces estrogen, while progesterone or testosterone production is extremely rare [6]. Estrogen production may result in menstrual abnormalities or postmenopausal bleeding and endometrial hyperplasia, progesterone production–decidualization of the endometrium, or peritoneum, while increased levels of testosterone result in amenorrhea or virilization [6]. Genetic predisposition is commonly associated with Peutz–Jeghers syndrome, a rare inherited dominant autosomal disease characterized by gastrointestinal polyps and hyperpigmentation of the skin and mucous membranes [7]. Owing to their rare prevalence and non-specific symptoms, the diagnosis of Sertoli cell ovarian tumors is difficult [6]. The final diagnosis is confirmed only after surgery by histological examination of the tumor [5].

Due to the substantially low incidence of ovarian Sertoli cell tumors, information on their clinical behavior, morphologic spectrum, optimal management, and prognosis is limited [6]. Herein, we report a patient of reproductive age who presented with complaints of amenorrhea for the past three years and was diagnosed with a left ovary Sertoli cell tumor with a small, undifferentiated, component-immature prepubertal-like Sertoli cell component. To the best of our knowledge, no similar cases have yet been published.

## 2. Case Report

A 29-year-old Caucasian woman (parity 0, abortions 0, and miscarriages 0) presented to a gynecologist with complaints of amenorrhea that had lasted for the past three years. Menarche occurred at 13 years old and menses was irregular. Hyperprolactinemia was found 8 months prior and pituitary prolactinoma was suspected. However, no pathological changes in the pituitary gland were observed after magnetic resonance imaging (MRI) of the head. Finally, polycystic ovary syndrome (PCOS) was diagnosed. Treatment of hyperprolactinemia with bromocriptine was effective. No familial cancer history was reported.

On physical examination, the patient’s body composition was normal and their body mass index (BMI) was 20.8 kg/m^2^. Visually, increased hair growth above the upper lip was observed. a slightly enlarged left adnexa was palpable during the bimanual pelvic examination.

**Figure 1:**
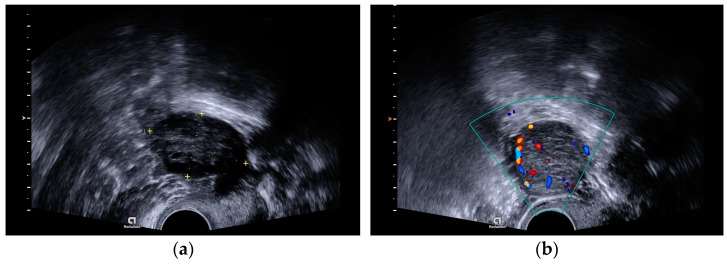
(**a**) A transvaginal ultrasound scan showing a polycystic structured ovary. (**b**) A transvaginal ultrasound scan showing a left ovary tumor with strong blood flow.

A transvaginal ultrasound scan revealed polycystic structured ovaries and a solid cystic formation of 32 × 31 mm size with strong blood flow in the left ovary (Figure 1a,b).

The laboratory tests indicated an elevated testosterone level (4.97 nmol/L), (normal ranges 0.48–1.85 nmol/L). Other hormones (progesterone and dehydroepiandrosterone sulfate (DHEAS-SO_4_)), sex hormone-binding globulin (SHBG), and the free androgen index (FAI) were within normal ranges. Ovarian tumor markers were conducted for the patient and they were within normal ranges (cancer antigen 125 (CA-125), 9.9 kU/L, and human epididymis protein 4 (HE4), 23.8 pmol/L). The patient was examined by an endocrinologist and a Synachtene test was performed. The result of the test was positive and congenital adrenal hyperplasia was suspected, so the patient was consulted by a clinical geneticist. Heterozygous deletion of CYP21A2 gene exons 1, 3, 4, 6, and 7 was identified by an multiplex ligation-dependent probe amplification (MLPA) assay. DNA sequencing confirmed the formation of a pathogenic combined gene (CYP21A1P(exons 1–8)/CYP21A2(exons 9–10)) in one allele of the gene, leading to a complete loss of the enzyme 21-hydroxylase’s activity. The second pathogenic variant in the CYP21A2 gene was not identified. The diagnosis of congenital adrenal hyperplasia was not confirmed, and hormonal changes were likely to be due to the ovarian tumor or PCOS.

It was decided to perform a laparoscopy. Inside the pelvic cavity, a solid yellowish tumor in the left ovary was found. There were no other visual changes in the abdominal cavity. During the operation, the ovarian tumor was removed, the left ovary was resected, and the abdominal cavity was washed and drained. The tumor was removed from the pelvic cavity without rupture. The contents of the tumor did not enter the peritoneal cavity.

**Figure 2:**
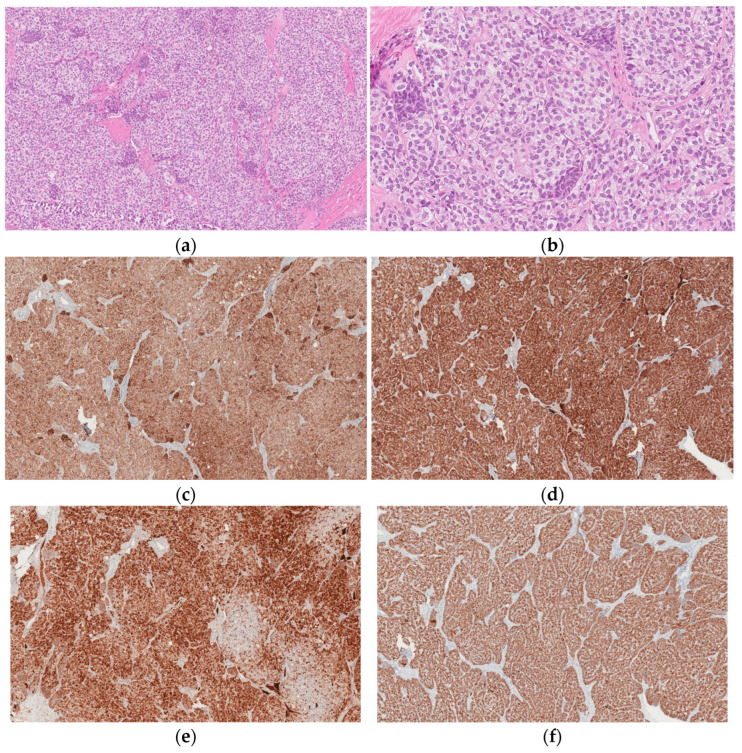
(**a**) Low-power view: solid tubules and trabecular structures composed of cells in hyalinized stroma with intervening small nests formed of cells with scant eosinophilic cytoplasm and hyperchromic, slightly irregular, and angular nuclei. (**b**) High-power view: small nests formed of cells with scant eosinophilic cytoplasm and hyperchromic, slightly irregular, and angular nuclei. (**c**) CD99. (**d**) Inhibin. (**e**) Calretinin. (**f**) AE1/AE3.

Histological examination revealed a left ovary Sertoli cell tumor with an immature prepubertal-like Sertoli cell component (Figure 2a–f). Upon microscopic examination, it was found that the tumor was formed from trabecular structures, solid tubules, or diffusely arranged cells in hyalinized stroma. Tumor cells were composed of moderate amounts of slightly eosinophilic cytoplasm with oval, slightly polymorphous nuclei. Focally, lipid-containing vacuoles and infrequent tubular structures were present. The tumor also contained scarce small nests (<5%) composed of cells with scant eosinophilic cytoplasm, hyperchromic, slightly irregular, and angular nuclei; there were four mitotic figures per ten high-power fields (HPFs).

No tumor cells were found in the cytological examination of the abdominal cavity fluid. After the operation, the patient’s general condition was satisfactory, and there were no complaints. Postoperative recovery was uneventful.

Due to the possible spread of the tumor, the patient was recommended to perform a left salpingo-oophorectomy, but the patient refused. Computed tomography (CT) of the neck, chest, abdomen, and pelvis was performed in the postoperative period, and no pathological changes were found.

Currently, it is one year after surgical treatment. The patient visits a gynecologist every 6 months. An ultrasound of the uterus and ovaries and an MRI of the pelvis were performed; no pathological changes were detected. The serum testosterone levels are within the normal range. The patient’s menstrual cycle became regular and the clinical symptoms of hyperandrogenism disappeared.

## 3. Discussion

According to the World Health Organization (WHO) classification, ovarian Sertoli cell tumors (OSCTs) are a subgroup of ovarian sex-cord stromal tumors [2]. OSCTs are rare; together with Sertoli–Leydig cell tumors and Leydig cell tumors, they account for less than 1% of all ovarian tumors [8]. They are most common in young patients (75% occur in those aged less than 30 years) and the onset age is between 2 and 79 years, with an average of about 30 years [5,8].

Ovarian Sertoli cell tumors (OSCTs) are associated with a relatively various morphological spectrum and heterogeneous endocrine behavior. Their detection may be either incidental in women seeking fertility or undergoing routine gynecological control due to associated hormonal hyperactivity, which requires endocrine evaluation [5]. The rarity of Sertoli cell tumors contributes to a low index of suspicion; therefore, thorough knowledge of the clinicopathological and immunological characteristics of such tumors is essential for diagnosis and proper management of the treatment and follow-up [5]. A distinctive feature of OSCTs is the lack of Leydig cells and immature stroma, which is in contrast to Sertoli–Leydig cell tumors [5].

According to the literature, endocrine manifestations occur in two-thirds of patients with ovarian Sertoli cell tumors [6]. Testosterone secretion and virilization are more common in Sertoli–Leydig or Leydig cell ovarian tumors [5]. Evidence of androgen secretion suggests the possible presence of unsampled Leydig cells in an otherwise pure Sertoli cell tumor, although pure Sertoli cell tumors seem to have this capacity [6]. In a clinicopathological study of patients with ovarian Sertoli cell tumors performed by Tavassoli et al., 4 out of 28 cases showed a virilizing effect, including hirsutism and amenorrhea [3]. In another, larger study performed by Oliva et al., 4 out of 54 patients had androgenic manifestations [6]. Our patient presented with complaints of prolonged amenorrhea, and laboratory tests revealed elevated testosterone levels. Following surgical treatment, their menstrual cycle became regular and their serum testosterone levels returned to normal.

In addition to a complete physical examination, the patient should undergo imaging tests that may include ultrasonography (US), CT, and MRI of the pelvis [5]. On US, OSCTs are echogenic solid masses, and on CT and MRI, they appear as encapsulated solid enhancing masses [4].

The final diagnosis of a Sertoli cell ovarian tumor is confirmed only after surgery by the histological examination of the tumor [5,6]. Macroscopically, Sertoli cell tumors are lobulated, solid, and yellow or brown tumors [6,8]. The histological hallmark of these ovarian tumors is hollow or solid closely packed tubules without or with only a few Leydig cells in the stroma [5,6,8,9]. Histologically, tumors have to be differentiated as endometrioid carcinoma and carcinoid [6]. According to the immunohistochemical study conducted by Oliva et al.., epithelial membrane antigen (EMA), inhibin, and chromogranin represent the most helpful triad of immunomarkers serving to exclude two common mimics of Sertoli cell tumors [6]. Although CD99, calretinin, and AE1/3 are frequently expressed in Sertoli cell tumors, they are less specific and not as helpful in differential diagnosis [6]. Severe cytological atypia and a high mitotic index (>5 per 10 HPF) have been observed to be indicators of unfavorable prognosis [9].

Most ovarian Sertoli cell tumors are found at stage I, and have a non-aggressive clinical course [5]. The primary treatment is unilateral oophorectomy [5,10]. According to the recommendations, lymphadenectomy should only be performed in cases of suspicious nodes upon imaging or intraoperative examination [10]. Because this type of tumor is more common in women of childbearing potential, for whom the preservation of fertility is particularly important, it is necessary to consider the future prognosis and choose the optimal treatment. For patients of reproductive age, fertility-sparing surgery that preserves the uterus and one ovary is safe in the early stages of the disease [5,10]. The need for adjuvant chemotherapy is assessed by the histological type and stage of the tumor, and if needed, the first choice option is usually a combination of BEP (bleomycin, etoposide, and cisplatin) [5,10]. According to the European Society of Gynaecological Oncology and the European Society for Paediatric Oncology (ESGO–SIOPE) guidelines, oophorectomy should be preferred to tumorectomy [10]. For our patient, the ovarian tumor was removed without rupture, the left ovary was resected, and postoperative CT revealed no residual pathology. Due to the possible spread of the tumor, the patient was recommended to perform a left salpingo-oophorectomy, but the patient refused.

Ovarian sex-cord stromal tumors are characterized by an indolent course and late recurrence (median time to relapse of 4–6 years) [2]. Therefore, long-term follow-up is recommended [2].

## 4. Conclusions

Sertoli cell tumors are relatively rare neoplasms of the ovary. The exact mechanism of ovarian Sertoli cell tumor development is still unclear. The rarity of Sertoli cell tumors contributes to a low index of suspicion; therefore, thorough knowledge of the clinicopathological and immunological characteristics of such tumors is essential for their diagnosis and proper management of the treatment and follow-up. Typically, they are unilateral, benign tumors of the ovary that are most commonly detected at an early stage in adolescents and young women of reproductive age. They are characterized by a wide variety of clinical manifestations, treated surgically, and, if diagnosed at an early stage, have good prognosis. As this type of tumor is more common in women of childbearing potential, for whom the preservation of fertility is important, it is necessary to consider the prognosis and choose the optimal treatment. For the patients of reproductive age, fertility-sparing surgery that preserves the uterus and one ovary is safe in the early stages of the disease.

## Data Availability

Not applicable.
